# Sequencing depth and genotype quality: accuracy and breeding operation considerations for genomic selection applications in autopolyploid crops

**DOI:** 10.1007/s00122-020-03673-2

**Published:** 2020-09-02

**Authors:** Dorcus C. Gemenet, Hannele Lindqvist-Kreuze, Bert De Boeck, Guilherme da Silva Pereira, Marcelo Mollinari, Zhao-Bang Zeng, G. Craig Yencho, Hugo Campos

**Affiliations:** 1 International Potato Center, ILRI Campus, P.O. Box 25171-00603, Nairobi, Kenya; 2 International Potato Center, Av. La Molina 1895, Lima, Peru; 3 North Carolina State University, Raleigh, NC 27695 USA; 4 Present Address: CGIAR Excellence in Breeding Platform, International Maize and Wheat Improvement Center (CIMMYT), ICRAF Campus, 1041-00621 Nairobi, Kenya; 5 Present Address: International Potato Center, Nairobi, Kenya

## Abstract

**Key message:**

Polypoid crop breeders can balance resources between density and sequencing depth, dosage information and fewer highly informative SNPs recommended, non-additive models and QTL advantages on prediction dependent on trait architecture.

**Abstract:**

The autopolyploid nature of potato and sweetpotato ensures a wide range of meiotic configurations and linkage phases leading to complex gene-action and pose problems in genotype data quality and genomic selection analyses. We used a 315-progeny biparental *F*_*1*_ population of hexaploid sweetpotato and a diversity panel of 380 tetraploid potato, genotyped using different platforms to answer the following questions: (i) do polyploid crop breeders need to invest more for additional sequencing depth? (ii) how many markers are required to make selection decisions? (iii) does considering non-additive genetic effects improve predictive ability (PA)? (iv) does considering dosage or quantitative trait loci (QTL) offer significant improvement to PA? Our results show that only a small number of highly informative single nucleotide polymorphisms (SNPs; ≤ 1000) are adequate for prediction in the type of populations we analyzed. We also show that considering dosage information and models considering only additive effects had the best PA for most traits, while the comparative advantage of considering non-additive genetic effects and including known QTL in the predictive model depended on trait architecture. We conclude that genomic selection can help accelerate the rate of genetic gains in potato and sweetpotato. However, application of genomic selection should be considered as part of optimizing the entire breeding program. Additionally, since the predictions in the current study are based on single populations, further studies on the effects of haplotype structure and inheritance on PA should be studied in actual multi-generation breeding populations.

**Electronic supplementary material:**

The online version of this article (10.1007/s00122-020-03673-2) contains supplementary material, which is available to authorized users.

## Introduction

Phenotyping under recurrent selection has been an important approach for variety development in plant breeding, with substantial success to date. However, this process may take a long time for most crops, particularly for clonally propagated crops (Slater et al. 2016). For example, in potato, it typically takes an entire year to develop enough tubers from botanical seed obtained from crossing nurseries, for experimental trial purposes. This is followed by at least 2 years of field evaluation for qualitative traits, with evaluation for most quantitative traits in replicated multi-environment trials beginning in around year four (Endelman et al. 2018). The same can be said for sweetpotato, although cycle times in sweetpotato are shorter by about a year due to the fact that the crop can be vegetatively propagated via stem cuttings (Grüneberg et al. 2009). This represents a stark contrast with what can be achieved in cereal and legume crops, where up to 6 generations can be raised within a calendar year (Watson et al. 2018), or in private corn breeding programs based in the USA and Europe which can raise multiple generations per year through the coordinated use of winter nurseries located in both hemispheres such as USA, Puerto Rico, Hawaii and Chile. This therefore implies that the estimation of parental value based on genetic designs and phenotypic evaluation in potato and sweetpotato increases the selection cycle time, thereby reducing the rate of genetic gains and the speed of delivery of superior, novel genetics to farmers.

The use of genetic markers for selection offers potential to reduce the breeding cycle time as selection can be done at an earlier stage. Previously proposed methods have involved identifying quantitative trait loci (QTL) via QTL mapping and genome-wide association studies (GWAS), but they have had little practical application in the actual development of new cultivars through plant breeding to date, especially for complex quantitative traits, since identifying the causal genes underlying QTL needed to make their application practical is costly (Xu and Crouch 2008). Genomic selection (GS) offers the ability to select parents within a shorter interval and increase selection intensity by predicting untested genotypes earlier while enhancing larger starting genetic variation. This approach uses genome-wide marker data to predict the performance of untested genotypes and estimate their breeding values (genomic estimated breeding values; GEBVs), based on a genotyped and phenotyped training population (Meuwissen et al. 2001). Genomic selection is emerging as the approach of choice to circumvent the limitations associated with use of QTL for marker-assisted selection and to improve the efficiency of phenotypic selection (Bernal-Vasquez et al. 2014). Good genetic progress can be made using GS, as long as factors that affect its predictive ability (PA), i.e., the correlation between phenotypic best linear unbiased estimators (BLUPs) and GEBVs, are well understood. These include trait architecture, the size of the training population, the relationship between the training and validation populations, heritability of the trait, the quality of phenotypic efforts, the level of linkage disequilibrium (LD), marker density, environmental variances and covariance among traits (Covarrubias-Pazaran et al. 2018).

The application of GS is taking shape in plant breeding with more and more crops exploring its utility (Spindel et al. 2016; Wang et al. 2018; Endelman et al. 2018; Covarrubias-Pazaran et al. 2018; Faville et al. 2018; Nyine et al. 2018; Bhandari et al. 2019). For crops like rice and wheat that are normally self-pollinated and have a high incidence of high-effect QTL (Spindel et al. 2016), faster success is expected from applying GS as prediction accuracy depends primarily on the factors listed above. However, breeders of autopolyploid, clonally propagated crops like potato and sweetpotato, which are normally heterogeneous and heterozygous, have to ask themselves additional questions and identify trade-off points that enhance the success of GS-assisted breeding (Slater et al. 2016; Endelman et al. 2018). Potato and sweetpotato present a wide range of meiotic configurations and linkage phases (Mollinari et al. 2020). In addition to causing complex gene-action effects, allelic and configuration diversity have consequences on genotyping and genotype data quality, which consequently affects downstream analysis for quantitative-genetic parameters required to make high-quality breeding decisions. Genotyping-by-sequencing (GBS) has currently become a genotyping method of choice in plant breeding (Poland and Rife 2012), but it is also prone to genotyping errors and a high level of missingness at low depth of sequencing, while high sequencing depth has additional cost implications. Data from polyploid crops are more prone to low-quality genotype calls at low sequencing depth when compared to diploid crops, because of uncertain allele dosages and possibility of non-random inheritance of alleles such as in preferential pairing or double reduction (Blischak et al. 2016, 2018).

Public sector breeding programs like those conducted in centers which are part of the Consultative Group on International Agricultural Research (CGIAR), and in the individual National Agricultural Research Systems (NARS) existing in many countries, are currently undergoing breeding program optimization efforts in order to keep up with the challenges of climate change and population increase (Cobb et al. 2019). Application of GS is one such tool for breeding program optimization. In order to develop GS tools to make more effective breeding efforts in autopolyploid crops such as potato and sweetpotato, we have taken a practical perspective within a plant breeding setting to address several pertinent questions related to application of GS in autopolyploids. We used real data sets from a 380 training-panel made up of advanced tetraploid potato clones and a 315-full-sib family (*F*_*1*_) of hexaploid sweetpotato, both developed by the International Potato Center (CIP) and genotyped using different platforms, to address the following questions: (i) do polyploid crop breeders need to invest more resources for additional sequencing depth? (ii) how many genetic markers are required to make selection decisions? (iii) does the consideration of non-additive genetic effects add value to predictive ability (PA) to enhance genetic gains either for population improvement or product development in polyploid crops? (iv) given the multiple alleles at loci with diverse meiotic configurations and linkage phases, does considering dosage, haplotypic or QTL effects offer significant improvement to PA to enhance genetic advances? We also discuss other factors that need to be considered while adopting GS as a decision support tool in an optimized breeding program.

## Materials and methods

### Genetic materials and phenotyping

#### Sweetpotato biparental population

A wide genetic variability exists in sweetpotato in terms of yield, nutritional content and culinary aspects, abiotic stress tolerance, biotic stress tolerance, among other attributes (Low et al. 2017). Introgression of high β-carotene content into locally adapted varieties is a major breeding objective especially in sub-Saharan Africa where vitamin A deficiency is prevalent. A 315-progeny full-sib family (*F*_*1*_) was developed by crossing a US-bred high β-carotene variety, ‘Beauregard,’ with an adapted, locally preferred, starchy, low β-carotene landrace variety, ‘Tanzania,’ at CIP—Peru. These two parents differ in additional traits of interest, and the population will henceforth be referred to as the BT population. The population was evaluated in six environments of Peru, for various quality-related and yield-related traits, between 2016 and 2017. The design was an 80 × 4 α-lattice with two–three replications, depending on location. The information about these trials is further described in the publications by Gemenet et al. (2020) and Pereira et al. (2020), and summaries of locations and experimental designs attached as Online Resource 1. Additionally, flesh color was evaluated in six environments of Uganda. The design was 80 × 4 α-lattice with three replications per location, in a 4.8 m^2^ plot size, with three locations over two years, as further described by Gemenet et al. (2020). The quality-related traits measured in the BT population include: dry matter (DM) content, measured as a percentage of the laboratory dried samples divided by the initial fresh weight of 100 g; Starch and β-carotene (BC) content, estimated using near-infrared reflectance spectroscopy (NIRS) and flesh color (FC), measured using internal color scales developed by CIP and partners. All quality-related traits were measured in Peru, but only flesh color was measured in Uganda (FC_U). Data are further described in Gemenet et al. (2020). For yield-related traits, total number of storage roots (TNR), number of commercial storage roots (NOCR), weight of total storage roots (RYTHA), weight of commercial storage roots (CYTHA) and total weight of foliage (FYTHA), were measured in the six experiments of Peru only. Data are further described in Pereira et al. (2020). Trait abbreviations are further defined in Table 1.

**Table 1:** Trait abbreviations and their description in sweetpotato and potato as used in the current study

Crop	Trait abbreviation	Trait description
Sweetpotato	DM	Dry matter content
	Starch	Starch content
	BC	Beta-carotene
	FC_P	Flesh color in Peru
	FC-U	Flesh color in Uganda
	NOCR	# Commercial storage roots
	TNR	# Total storage roots
	CYTHA	Commercial storage root weight
	RYTHA	Total storage root weigh
	FYTHA	Total foliage yield weight
Potato	LB2014_O	Late blight in 2014 in Oxapampa, Peru
	LB2016_Y	Late blight 2016 in Yunnan, China
	PVY_L	Potato virus Y in Lima, Peru
	AYP_K	Average yield per plant in Kunming, China
	WMT_K	Weight of marketable tubers in Kunming, China
	TTW16_Ica	Total tuber weight in 2016 in Ica-Peru
	TTW16_HLJ	Total tuber weight in 2016 in Heilongjiang, China

The quality-related traits were analyzed by fitting the following linear mixed model in ASREML:

1 $$y_{ijkl} = \mu + g_{i} + e_{l} + r_{k\left( l \right)} + b_{jk\left( l \right)} + (ge_{i} )_{l} + \varepsilon_{ijk\left( l \right)}$$

where $y_{ijkl}$ = the vector of phenotypes of genotype *i* in block *j* within replicate *k* of environment *l*, *µ* = population mean, $g_{i}$ = the fixed treatment (genotype) effect, $e_{l}$ = the random effect of environment *l*, $r_{k\left( l \right)}$ = random effect of replicate *k* in environment *l,* $b_{jk\left( l \right)}$= random effect of block *j* within replicate *k* of environment *l,* $(ge_{i} )_{l}$= random effect of genotype *i* in environment l (l = 1,..,*L*), $\varepsilon_{ijk\left( l \right)}$ = random error of the residuals, assuming $e_{l} \sim N\left( {0,\sigma_{e}^{2} } \right)$, $r_{k\left( l \right)} \sim N(0,\sigma_{r\left( l \right)}^{2} )$, $b_{jk\left( l \right)} \sim N\left( {0,\sigma_{b\left( l \right)}^{2} } \right)$, $ge_{i} \sim N(0,\left. \sum \right)$ with ∑ = variance–covariance matrix of the genotypes across *L *= *5* environments, allowing for heterogeneity of genetic variances and covariances across environments. The best fitting model was chosen by Akaike’s information criterion (AIC) and differed slightly for the different traits. For DM, Starch and FC_U, a factor analytic model (Piepho 1998) of order 1 was used, and for BC a factor analytic model of order 2, to model the variance covariance matrix ∑. For FC, an unstructured variance–covariance matrix ∑ was used in the final model. The random error of residuals was assumed as $\varepsilon_{ijk\left( l \right)} \sim N\left( {0,\sigma_{l}^{2} } \right)$ (Gemenet et al. 2020).

The yield-related traits were also analyzed with linear mixed models as described by Pereira et al. (2020) using restricted maximum likelihood (REML) in GENSTAT 14 as:

2 $$y_{ijkl} = \mu + g_{i} + e_{l} + r_{k\left( l \right)} + b_{jkl} + ge_{il} + \varepsilon_{ijkl}$$

where $y_{ijkl}$ = the vector of phenotypes as above, $\mu =$ population mean, $g_{i} =$ the fixed treatment (genotype) effect, $e_{l}$ = fixed effect of environment *l*, $r_{k\left( l \right)}$ = fixed effect of replication *k* in environment *l*, $b_{jkl}$ = random effect of block *j* within replication *k* in environment *l*; $b_{jkl} \sim N\left( {0,\sigma_{b}^{2} } \right)$, $ge_{il}$ = the fixed interaction effect of genotype *i* and environment *l*, and $\varepsilon_{ijkl} \sim N\left( {0,\sigma^{2} } \right)$ is the random residual error. The two data classes (quality-related traits and yield-related traits) were analyzed using different methods because the two analysts made different assumptions regarding variance components and genotype-by-environment interaction. The models for yield-related traits assumed compound symmetry and fixed correlation among environments, which may be less realistic in estimating the covariance structure of the different traits. This could lead to poor estimates of standard errors of differences between some means and hence some difference in adjusted means. However, such differences are not expected to significantly affect the findings from further analyses using these adjusted means in the current study. The best linear unbiased estimators (BLUEs) as obtained by fitting the above models to the experimental data with genotypes as fixed were then used to estimate GEBVs.

#### Potato trait observation network population

A 380-genotype panel made up of advanced clones from the potato breeding program and representing all breeding populations at CIP was assembled for a trait observation network (TON) in Peru, China and Ethiopia. Henceforth, we shall refer to this population as the TON panel. The evaluation of the panel was carried out in diverse agro-ecological zones, and in subsets of genotypes subject to participating NARS’ partner capacity and/or ability to produce enough mini-tubers for experimentation. The experimental sites, experimental designs and the number of genotypes evaluated per experiment are summarized in Table 2. The TON panel was evaluated for maturity (bulking) by tuber characteristics at three harvest dates where average yield per plant (kg; AYP) and weight of marketable tubers per plant (kg; WMT) were measured. Additionally, mature tuber weight was evaluated by measuring total tuber weight per plant (TTW; kg). In Peru, TTW was measured as the average total tuber weight across three drought-related treatments: terminal drought (irrigation stopped at flowering until harvest; TTW16_TD), recovery (partially irrigated after drought stress; TTW16_REC) and fully irrigated (normally irrigated throughout the growth period; TTW16_NI), while random drought was used in China, with no controlled treatments. Resistance to potato virus Y (PVY) was evaluated after infection with virulent vectors and susceptible spreader rows using standard protocols at CIP, while late blight resistance (LB) was evaluated by growing the population in endemic disease pressure and scored using standard protocols at CIP. Trait abbreviations are defined in Table 1.

**Table 2:** Locations, designs and traits measured in the trait observation network (TON) panel of potato

Country	Location	Agroecology
Peru	Lima, La Molina 12.0820° S, 76.9282° W	Lowland sub-tropics
	Ica, Ica 14.0755° S, 75.7342° W	
	Pasco, Oxapampa 10.5853° S, 75.4053° W	Highland tropics
China	Yunnan, Kunming 24.8801° N, 102.8329° E	
	Mixed agriculture systems, lowland & highland	
	Heilongjiang, Harbin 45.8038° N, 126.5350° E	Temperate (long day)

Trait group	Trait	Location, country, year	Trial design	#Genotype
Late blight resistance	LB2014_O	Oxapampa, Peru, 2014	RCBD	241
	LB2016_Y	Yunnan, China, 2016	RCBD	336
Virus resistance	PVY_L	Lima, Peru, 2016–2018	RCBD	341
Bulking-based maturity	AYP_K	Kunming, China, 2016	RCBD	317
	WMT_K	Kunming, China, 2016	RCBD	317
Mature tuber weight	TTW16_Ica	Ica, Peru, 2016	Augmented	269
	TTW16_HLJ	Heilongjiang, China, 2016	Augmented	300

Unlike in sweetpotato where phenotype and genotype data were balanced across experiments, (292 + Parents for DArTSeq and 315 + parents for GBSpoly), the potato experiments were unbalanced in terms of experimental genotypes. For the purposes of this study, we only selected the locations with the highest training population per trait. Consequently, we used AYP from Kunming (China; AYP_K), WMT from Kunming (China; WMT_K), LB from Oxapampa (Peru; LB2014_O), LB from Yunnan (China; LB2016_Y), PVY from Lima (Peru; PVY_L), TTW averaged across three treatments of 2016 in Ica (Peru; TTW16_Ica) and TTW in 2016 from Heilongjiang (China; TTW16_HLJ), all having number of genotypes indicated in Table 2. The experiments were analyzed as single trials, depending on the experimental design used as summarized in Table 2. A linear mixed model, taking into account the respective experimental design, was fitted to the phenotypic data. For those traits with different treatments like TTW in Peru, the joint adjusted means were additionally obtained across all treatments by fitting a linear mixed model. Genotype was considered as a fixed effect in these mixed models, so that BLUEs for the genotypic means were obtained for each trait and used to predict GEBVs.

### Genotyping and variant calling

The full 315-progeny of the BT (sweetpotato) population was genotyped together with the parents using an optimized protocol for hexaploid sweetpotato, ‘GBSpoly’ at North Carolina State University (NCSU). Additionally, a subsample of 292-progeny and the two parents of the BT population were genotyped by DArTSeq™ in Australia, under the collaboration between the Integrated Genotyping Service and Support (IGSS) platform at the Biosciences east and central Africa (BecA) hub in Nairobi, Kenya and DArT. The 380 genotypes of the TON population (potato) were genotyped by GBS at Cornell University.

#### DArTSeq™ for Sweetpotato

DArTseq™ represents a combination of DArT complexity reduction methods and next-generation sequencing platforms (Kilian et al. 2012; Courtois et al. 2013; Raman et al. 2014; Cruz et al. 2013). Therefore, DArTseq™ represents a new implementation of sequencing complexity reduced representations (Altshuler et al. 2000) and more recent applications of this concept on the next-generation sequencing platforms (Baird et al. 2008; Elshire et al. 2011). Similar to previous DArT methods based on array hybridizations, the technology is optimized for each organism and application by selecting the most appropriate complexity reduction method (both the size of the representation and the fraction of a genome selected for assays). Four methods of complexity reduction were tested in sweetpotato (data not presented), and the *PstI*-*MseI* method was selected. DNA samples were processed in digestion/ligation reactions principally as per Kilian et al. (2012) but replacing a single *PstI*-compatible adaptor with two different adaptors corresponding to two different restriction enzyme (RE) overhangs. The *PstI*-compatible adapter was designed to include Illumina flowcell attachment sequence, primer sequence and ‘staggered,’ varying length barcode region, similar to the sequence reported by Elshire et al. (2011). This reverse adapter contained a flowcell attachment region and a *MseI*-compatible overhang sequence. Only ‘mixed fragments’ (*PstI*-*MseI*) were effectively amplified in 30 rounds of PCR using the following reaction conditions: (i) 94 °C for 1 min, (ii) 30 cycles of: 94 °C for 20 s, 58 °C for 30 s, 72 °C for 45 s and (iii) 72 °C for 7 min. After PCR, equimolar amounts of amplification products from each sample of the 96-well microtiter plate were bulked and applied to c-Bot (Illumina) bridge PCR followed by sequencing on Illumina Hiseq 2000. The sequencing (single read) was run for 77 cycles. Sequences generated from each lane were processed using proprietary DArT analytical pipelines. In the primary pipeline, the FastQ files were first processed to filter away poor-quality sequences, applying more stringent selection criteria to the barcode region compared to the rest of the sequence. This was to ensure reliability in the assignments of the sequences to specific samples carried in the ‘barcode split’ step. Approximately 2,000,000 sequences per barcode/sample were identified and used in marker calling. Finally, identical sequences were collapsed into ‘fastqcoll files.’ The fastqcoll files were ‘groomed’ using DArT PL’s proprietary algorithm which corrects low-quality base from singleton tag into a correct base using collapsed tags with multiple members as a template. The ‘groomed’ fastqcoll files were used in the secondary pipeline for DArT PL’s proprietary SNP and SilicoDArT (presence/absence of restriction fragments in representation) calling algorithms (DArTsoft14). For SNP calling, all tags from all libraries included in the DArTsoft14 analysis were clustered using DArT PL’s C ++ algorithm at the threshold distance of 3, followed by parsing of the clusters into separate SNP loci using a range of technical parameters, especially the balance of read counts for the allelic pairs. Additional selection criteria were added to the algorithm based on analysis of approximately 1000 controlled cross populations. Testing a range of tag counts parameters facilitated selection of true allelic variants from paralogous sequences. In addition, multiple samples were processed from DNA to allelic calls as technical replicates and scoring consistency was used as the main selection criteria for high-quality/low error rate markers. Calling quality was assured by high average read depth per locus (> 30X). The SNPs were coded as 0 = AA, 1 = BB, 2 = AB and ‘-’= Missing. The sequences were not aligned to a reference genome because by the time of genotyping, the diploid references (Wu et al. 2018) had not been published.

#### GBSPoly© for Sweetpotato

GBSpoly is an optimized protocol for hexaploid sweetpotato developed at NCSU as part of a project focusing on developing genomic tools for sweetpotato improvement. The DNA was checked for quality on 1% agarose gel and quantified based on the PicoGreen florescence-based assay and the concentration was normalized to 50 ng/µl. Initially, several optimization efforts regarding restriction enzyme pairing were carried out (data not shown) and *CviAII*-*TseI* was selected to be the best combination for hexaploid sweetpotato. Therefore, 1 µg of DNA was double-digested using five units of *CviAII* for three hours at 25 °C followed by digestion with *TseI* for another three hours at 65 °C. A new England Biolabs (NEB) CutSmart buffer was used to make up a total volume of 30 µl. Purification of the digested samples was done using AMPure XP magnetic beads from ThermoFisher™ and quantified with PicoGreen assay. Barcodes were designed to account for substitution and indel errors and had an 8-bp buffer sequence to ensure that the barcode lay within high-quality base call regions of the sequence reads. Additional double digests on 64-plex pooled samples, purification and size selection steps were carried out as described by Wadl et al. (2018) before performing 125 bp single-end sequencing on a total of 40 sequencing lanes (8 lanes for each of the 5 libraries) of the Illumina HiSeq 2500 platform. The resultant FastQ files were aligned to reference genomes of two wild relatives of sweetpotato, *Ipomoea trifida* and *Ipomoea triloba* (Wu et al. 2018), and variant calling done using the GBSapp pipeline as described by Wadl et al. (2018). The SNPs were coded according to the dosage of the alternative allele as 0 = AAAAAA, 1 = AAAAAB, 2 = AAAABB, 3 = AAABBB, 4 = AABBBB, 5 = ABBBBB, 6 = BBBBBB. The variant calling process is summarized in Online Resource 2.

#### GBSCornell for potato

The 380-genotype TON panel was genotyped by Cornell University using GBS in 2015. The DNA was digested with *EcoT221* restriction enzyme, and 48-plex libraries were prepared for sequencing, using customized GBS protocols at Cornell. The resultant FastQ files were quality controlled and variant calling done using GATK HaplotypeCaller option (Poplin et al. 2017), disabling the duplicate read filter (this is recommended for GBS data) and using the joint genotyping -ERC GVCF mode, as further described in Lindqvist-Kreuze et al. (2020). The reads were aligned to the potato genome reference sequenced from *S. tuberosum* group Phureja, line DM1-3 516 R44, a doubled monoploid (DM) via anther culture by the potato genome sequencing consortium (PGSC). Version PGSC_DM_v4.03 of the reference genome was used in alignment. The barcodes were removed using stacks, and the ends were trimmed using trim-galore, followed by mapping to the reference using BWA. Resultant SAM files were processed using samtools and variants called using GATK Haplotype caller, targeting biallelic SNPs only. The SNPs were coded according to the dosage of the alternative allele as 0 = AAAA, 1 = AAAB, 2 = AABB, 3 = ABBB and 4 = BBBB. The SNP filtering was done using bcftools allowing only for those SNPs with MAF of ≥ 3%, call rate of ≥ 70%, average genotype quality (GQ) ≥ 30 and minimum read depth (DP) ≥ 16 (Lindqvist-Kreuze et al. 2020).

Although estimating allele frequencies in polyploids may encounter many challenges as explained by De Silva et al. (2005), allele frequencies for the polyploid data (‘GBSpoly’ for sweetpotato and ‘GBSCornell’ for potato) in the current study were estimated by counting the number of alleles in each dosage-based genotype, since quantitative genotyping was used for both methods.

### Model comparison for predictive ability

We used the AGHmatrix package (Amadeu et al. 2016) to develop kinship G-matrices partitioning genetic variation based on several gene-action models. For the BT population DArTSeq markers (sweetpotato) where we did not have dosage information, we developed an additive G-matrix according to VanRaden (2008), herein referred to as Add_2x_DArTseq, and a non-additive-effects G-matrix according Vitezica et al. (2013), herein referred to as NonAdd_2x_DArTSeq. For the BT population GBSpoly (sweetpotato) and TON population GBSCornell (potato) data where we had dosage information, we employed three models to develop the G-matrices: (i) modeling only additive effects, according to VanRaden (2008) herein referred to as Add_6x_GBSpoly for sweetpotato and Add_4x_GBSCornell for potato, (ii) modeling additive plus non-additive effects, according to Slater et al. (2016) herein referred to as Add + Non_6x_GBSpoly for sweetpotato and Add + Non_4x_GBSCornell for potato and (iii) a pseudo-diploidized effect model according to Slater et al. (2016), herein referred to as Pseudo_2x_GBSpoly for sweetpotato and Pseudo_2x_GBSCornell. The pseudo-diploidization collapses all dosage classes between the nulliplex and the hexaplex (in sweetpotato), and between the nulliplex and tetraplex (in potato) into one heterozygous class, under the assumption that all heterozygotes have an equal effect which falls in between both homozygotes. In the case of potato, the design matrix coding for the pseudo-diploid, additive autotetraploid and full autotetraploid was as described by Slater et al. (2016), while that for sweetpotato is shown in Table 3. During kinship matrix development, additional filters were applied to the genotype data, to have minimum allele frequency (MAF) ≥ 30%, and call rate ≥ 90%. We used genomic best linear unbiased prediction (G-BLUP; Clark and van der Werf 2013) to compare the predictive ability (PA) of the five models for sweetpotato and three models for potato using the kinship matrices as variance–covariance matrices to fit the compressed linear mixed model (Zhang et al. 2010) and estimate genomic best linear unbiased predictors (G-BLUPs). The software GAPIT (Lipka et al. 2012) was used in the G-BLUP prediction fitting the following general model:

3 $$y = 1_{n} \mu + Zu + e$$

where *y* = vector of phenotypic data, *1*_*n*_ is the vector of ones, *μ* = population mean, *Z* = the known design matrix for genotypes, *u* = random genetic effects and ~ $N\left( {0,\sigma_{a}^{2} K or \sigma_{a + na}^{2} K} \right)$ with *K* = kinship matrix, *a* = additive model, *na* = non-additive model, *e* = vector of residuals ~ $N\left( {0, \sigma_{e}^{2} I} \right)$.

**Table 3:** Proposed design matrix coding for auto-hexaploid sweetpotato as adapted from Slater et al. 2016

Effects/marker	Pseudo_2x	Add_6x	Add + Non_6x						
	1	1	1	2	3	4	5	6	7
AAAAAA	0	0	1	0	0	0	0	0	0
AAAAAB	1	1	0	1	0	0	0	0	0
AAAABB	1	2	0	0	1	0	0	0	0
AAABBB	1	3	0	0	0	1	0	0	0
AABBBB	1	4	0	0	0	0	1	0	0
ABBBBB	1	5	0	0	0	0	0	1	0
BBBBBB	2	6	0	0	0	0	0	0	1

Cross-validation was done by randomly setting 20% of the population to missing phenotypes to be used as a validation set. We used 1000 iterations (replications) to estimate the predictive ability of the models using both simple/oligo traits (quality traits in sweetpotato, disease traits in potato) and complex traits (storage root or tuber yield and yield component traits in both), as defined in Table 1.

The PA was calculated as Pearson’s correlation between the observed BLUPs and the genome estimated breeding values (GEBVs). Differences in PA among models per trait were tested using a simple one-way analysis of variance with models as factor. The correlation coefficients per replication were Fisher Z-transformed and means compared on these Z values using a one-way ANOVA with models as factor. The average PA was then obtained by back transforming the average of the Z values. Quantitative-genetic parameters were tested for the additive model with or without dosage by obtaining the additive genetic variation $(\sigma_{a}^{2}$) and random residual effects $\left( {\sigma_{e}^{2} } \right)$ from the mixed linear model and calculating narrow-sense heritability (*h*^*2*^) for each trait as:

4 $$h^{2} = \frac{{\sigma_{a}^{2} }}{{\left( {\sigma_{a}^{2} + \sigma_{e}^{2} } \right)}}$$

Additionally, we calculated the estimated rate of genetic gains from genomic selection per additive model with or without dosage for each trait according to Oliveira et al. (2019) as:

5 $$\Delta GG = \frac{{\left( {i*\sigma_{a} *PA} \right)}}{L}$$

where $\Delta GG =$ rate of genetic gains, $i =$ selection intensity, $\sigma_{a} =$ square root of additive genetic variation, $PA =$ predictive ability and $L =$ length of breeding cycle, assuming *L *= 5 for sweetpotato following the accelerated breeding scheme currently implemented (Mwanga et al. 2017), and *L *= 8 for potato.

### How many markers are adequate for prediction?

For the sweetpotato *F*_*1*_ population, we used the original GBSpoly data, using different filtering criteria to end up with different number of markers. We used three criteria (i) total number of SNPs filtered at 10% MAF and ≥ 90% call rate, (ii) total number of SNPs filtered at 30% MAF and ≥ 90% call rate (used in the analyses above) and (iii) a random sample of 15,000 SNPs from the total number of SNPs and filtered at 30% MAF and ≥ 90% call rate. In potato, the total number of SNPs was filtered using two criteria: (i) 30% MAF and ≥ 90% call rate, (ii) 40% MAF and ≥ 90% call rate. Predictions were carried out for all traits measured using these criteria. To separate the effects of allele frequency from the effects of number of markers on PA, we also used the original GBSpoly data in sweetpotato, filtered at constant MAF and randomly sampled different number of markers, which we used to compare PA in one quality-related simple trait (β-carotene; BC) and one yield-related complex trait (total number of storage roots; RYTHA). We used 10, 000, 5000, 1000 and 500 SNPs, all filtered to MAF ≥ 5%. The model considering only additive effects (Add_6x_GBSpoly) was used in comparing the effect of allele frequency and number of markers in sweetpotato, while all three models were tested between the two filtering criteria in potato.

### Incorporating haplotypic-QTL in prediction models for sweetpotato

By taking advantage of the fully phased integrated linkage map from BT (Mollinari et al. 2020), we tested the predictive ability from QTL-informed models. To achieve this, we used the same cross-validation scheme as above, where 80%:20% random samples were used as training and testing populations, respectively, replicated 1000 times. In order to detect QTL, we ran our random-effect multiple interval mapping (REMIM) using a sequential forward search (Pereira et al. 2020). Using the sequential forward search, we used score statistics to test map positions every 2 centiMorgans (cM) and added QTL to the random-effect model, one QTL at a time, using a relaxed genome-wide significance level threshold (α = 0.20). A window size of 20 cM was used to avoid selection of another position very close to a QTL already in the model. For G-BLUP models, realized kinship matrices were based on the haplotype information from markers positioned every 2 cM in the genetic map. For QTL-BLUP (Q-BLUP), realized kinship matrices were based on the haplotypes from QTL-peak marker; if there were more than one QTL, their kinship matrices were averaged out; if there were no QTL, we obtained the prediction as in G-BLUP. For Q + G-BLUP models, two terms were fitted, each with realized kinship matrices based on QTL-peak markers (like for Q-BLUP) and the remaining markers in the linkage map except those selected as QTL.

## Results

### SNP profiles from the genotyping platforms

**Fig. 1:**
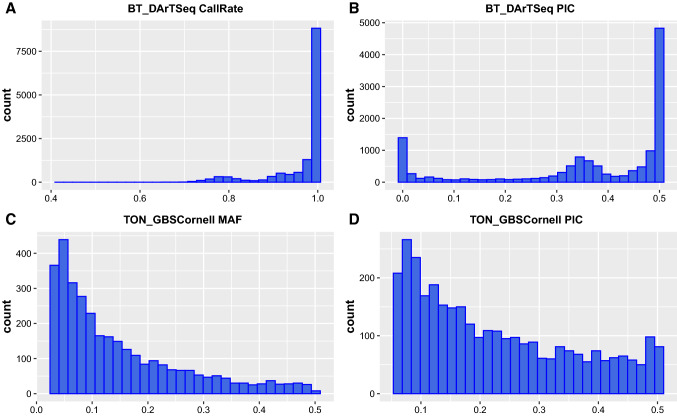
Quality attributes of the SNP profiles from DArTSeq (call rate (**a**) and polymorphic information content; PIC (**b**)) data in sweetpotato and GBSCornell (minor allele frequency; MAF (**c**) and PIC (**d**)) in potato. The y-axes in all plots refer to the number of SNPs

DArTseq sequencing of sweetpotato resulted in 13,504 biallelic SNPs (Online Resource 3). The call rates and polymorphic information content (PIC) are shown in Fig. 1a, b and ranged from about 0.4 (40%)–1.0 (100%), with a mean of 0.96 (96%) for call rate and from 0 to 0.5 with a mean of 0.37 for PIC. Stringent filtering at a call rate ≥ 80% and PIC ≥ 0.25 left 9649 SNPs that were used in AGHMatrix. Additional filtering in AGHMatrix at ≥ 80% call rate and ≥ 30% MAF resulted in 6015 diploidized, biallelic SNPs being used to develop the matrices following additive (Add_2x_DArTSeq) and non-additive (NonAdd_2x_DArTSeq) models.

Cornell GBS in potato resulted in 295,401 biallelic SNPs at the variant calling step that were then hard-filtered to 3262 high confidence SNPs by setting MAF ≥ 3%, call rate ≥ 70% and average read depth (DP) ≥ 16 (Online Resource 4). The 3262 SNP profiles are shown in Fig. 1C, D showing MAF ranging from 0.03 (3%) to 0.5 (50%), with a mean of 0.15 (15%) and PIC ranging from 0.0 to 0.5, with a mean of 0.23. The 3262 SNPs were used in the AGHMatrix relationship matrix development. For a relative comparison of models across crops for trait groups, we also filtered the Cornell GBS data in AGHMatrix at ≥ 90% call rate and ≥ 30% MAF as done for DArTSeq data above, which resulted in 411 SNPs used to develop the additive (Add_4x_GBSCornell), additive plus non-additive (Add + Non_4x_GBSCornell) and the pseudo-diploidized (Pseudo_2x_GBSCornell) models. Examining the relationship matrices indicated that at MAF ≥ 30%, the full model (Add + Non_4x_GBSCornell) was mainly monomorphic. For potato therefore, we also changed the MAF to ≥ 40%, which resulted in 178 SNPs that were used to develop a second set of relationship matrices. All PA comparisons among traits for potato are based on this matrix (MAF ≥ 40%).

**Fig. 2:**
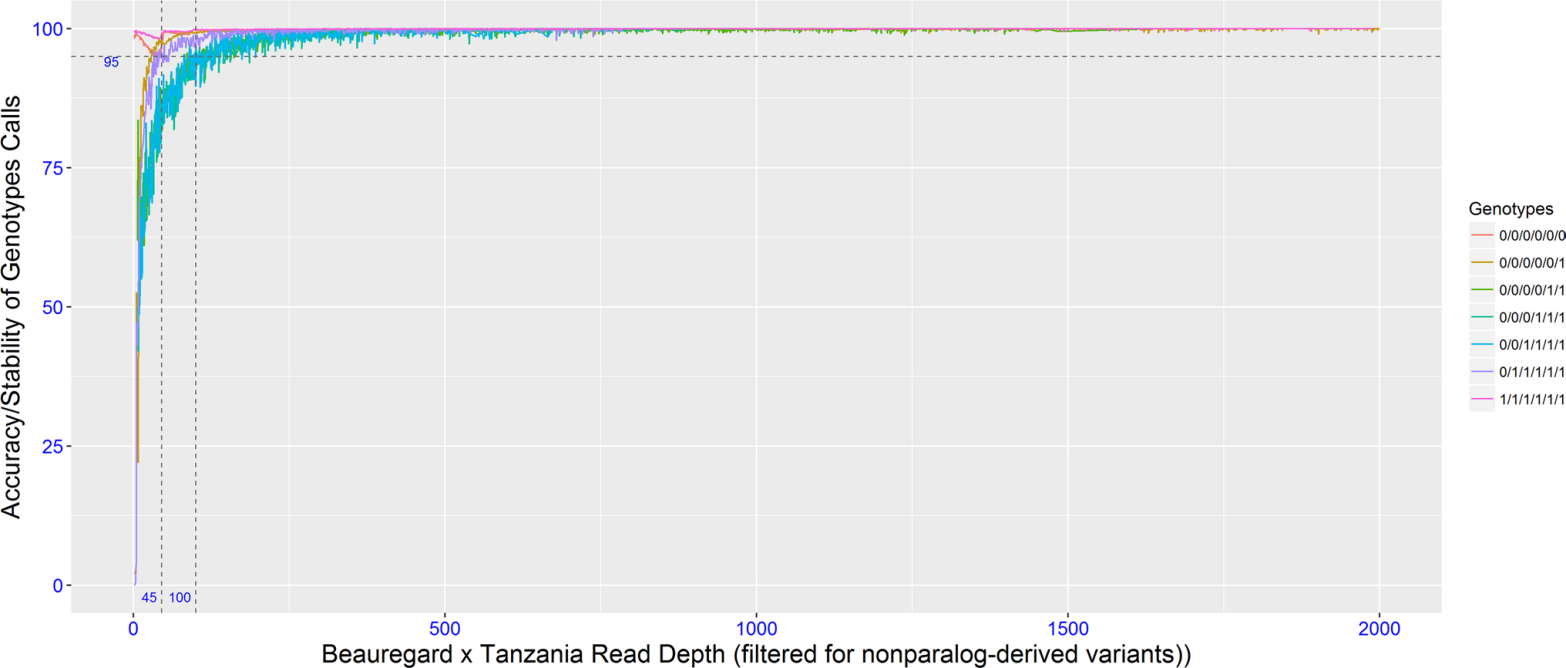
Paralog filtering and empirical estimation of read depth threshold for high-fidelity SNPs in sweetpotato. The plot shows stability of each genotypic classes (nulliplex to hexaploid) with increasing read depth, which is achieved by resampling reads (without replacement) to simulate Illumina sequencing of each locus and at incremental read depths. SNPs derived from paralogs or repetitive sequences were eliminated due to low stability even at high read depths (more details in Wadl et al. 2018)

**Fig. 3:**
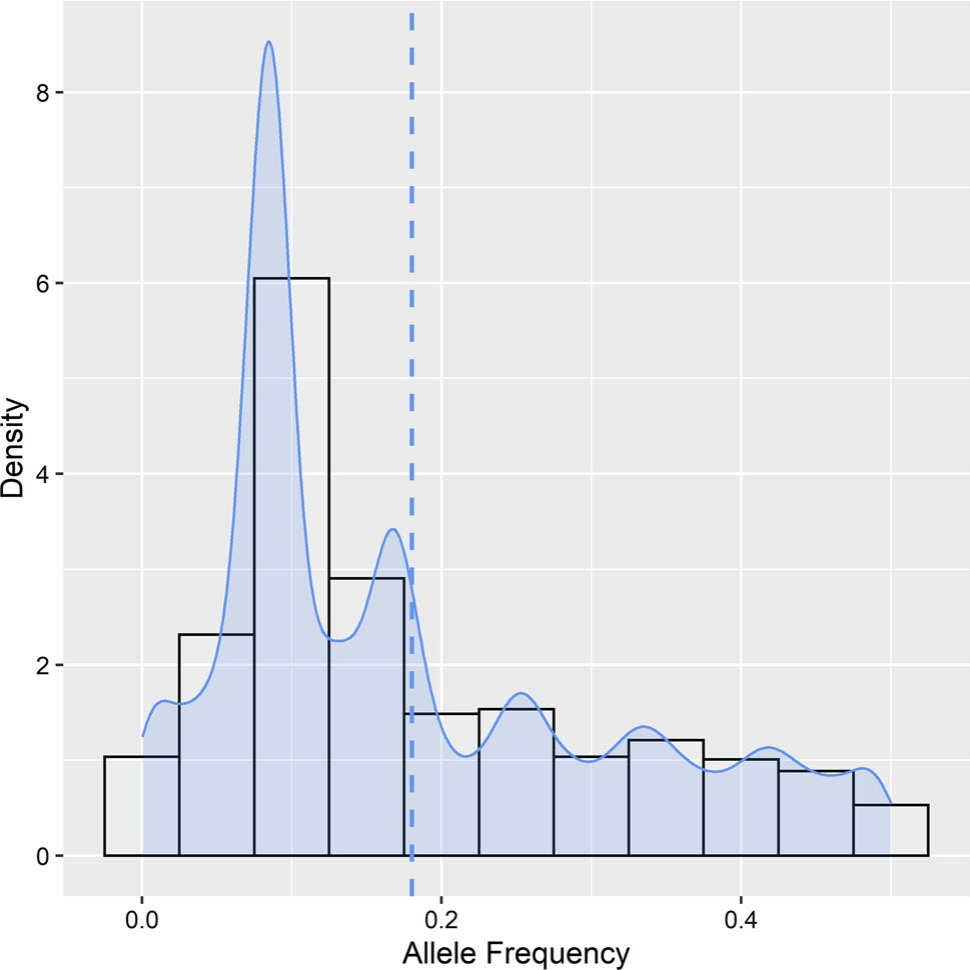
Allele frequency density plot for GBSpoly data in sweetpotato

For GBSpoly in sweetpotato called according to Wadl et al. (2018), the empirical estimation of read depth threshold to ensure high-fidelity SNPs was carried out after paralog filtering (Fig. 2). The stability of each genotypic classes (nulliplex to hexaploid) was evaluated with increasing read depth, which was achieved by resampling reads (without replacement) to simulate Illumina sequencing of each locus at incremental read depths. SNPs derived from paralogs or repetitive sequences were eliminated due to low stability even at high read depths (more details in Wadl et al. 2018). Figure 3 shows the allele frequency of the GBSpoly data ranging from 0 to 50%, while expected segregation ratios in hexaploid sweetpotato as well as genotype distribution in each segregation class after filtering for segregation distortion at unmethylated loci are provided as Online Resource 5. Consequently, for sweetpotato, GBSpoly data were filtered to this high depth of coverage, with MAF ≥ 5%. This resulted in 34,390 high confidence SNPs (Online Resource 6) that were used in AGHMatrix to develop the additive (Add_6x_GBSpoly), additive plus non-additive (Add + Non_6x_GBSpoly) and the pseudo-diploidized (Pseudo_2x_GBSpoly) relationship matrices. The filters in AGHMatrix were set to ≥ 90% call rate and ≥ 30% MAF as for the preceding data types and resulted in a final 2883 SNPs that developed the matrices for model comparison. For comparing the combined effects of number of markers and allele frequency on PA, the first filtering criteria of 10% MAF and ≥ 90% call rate resulted in 10,358 SNPs, while the third criteria based on a random sample of 15,000 SNPs resulted in 1291 SNPs at ≥ 30% MAF and ≥ 90% call rate that were used in PA comparison, based on the model considering additive effects only (Add_6x_GBSpoly). To examine the effect of markers only, without the compounding effect of allele frequency, 500, 1000, 5000 and 10,000 SNPs were randomly selected from the original 34,390 SNPs all at MAF ≥ 5% and tested using the model considering additive effects only (Add_6x_GBSpoly).

The comparison of models with(out) QTL and use of markers per se or haplotypes was carried out only in sweetpotato, using 30,684 SNPs from the same genotyping platform and data set, filtered and processed as described by Mollinari et al. (2020), which were used to develop a 2708.4 cM phased genetic linkage map for sweetpotato, and subsequent QTL analyses (Pereira et al. 2020; Gemenet et al. 2020). The QTL summary tables in sweetpotato as previously reported by Pereira et al. (2020), Gemenet et al. (2020) are provided as Online Resource 7. Sweetpotato BLUEs are provided in Online Resource 8, while potato BLUEs are provided as Online Resource 9.

### Genotyping platforms, genetic effects and predictive ability

**Fig. 4:**
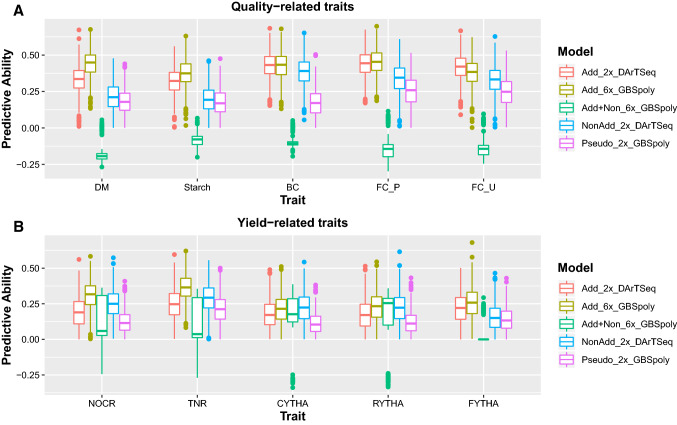
Model comparison in sweetpotato. Boxplots comparing predictive ability of additive-effects-only models without dosage (Add_2x_DArTseq) and with dosage (Add_6x_GBSpoly); models considering also non-additive effects (NonAdd_2x_DArTSeq; Add + Non_6x_GBSpoly); and pseudo-diploidized dosage data (Pseudo_2x_GBSpoly) for quality-related traits (A; DM = dry matter, starch, BC = β-carotene, FC_P = flesh color in Peru; FC_U = flesh color in Uganda); and yield-related traits (B; NOCR = number of commercial storage roots, TNR = total number of storage roots, CYTHA = weight of commercial storage roots, RYTHA = weight of total storage roots, FYTHA = total weight of foliage) in a full-sib family of sweetpotato

**Fig. 5:**
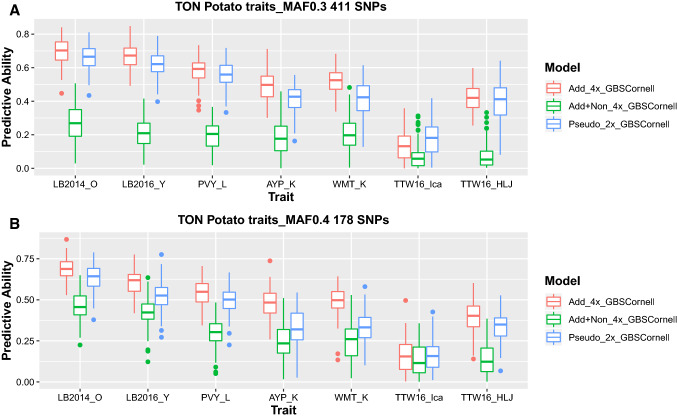
Model comparison in potato. Box plots comparing predictive ability of additive-effects-only model (Add_4x_GBSCornell); additive and non-additive effects (Add + Non_4x_GBSCornell); and pseudo-diploidized dosage data (Pseudo_2x_GBSCornell); using minimum allele frequency (MAF) ≥ 30% (A; 411 SNPs) and MAF ≥ 40% (B; 178 SNPs). LB2014_O = late blight in Oxapampa (Peru) in 2014, LB2016_Y = late blight in Yunnan (China) in 2016, PVY_L = potato virus Y in Lima (Peru), AYP_K = average yield per plant in Kunming (China), WMT_K = weight of marketable tubers in Kunming, TTW16_Ica = total tuber weight in Ica (Peru) in 2016 across three drought treatments, TTW16_HLJ = total tuber weight in Heilongjiang (China) in 2016, single treatment, in potato

In sweetpotato, the diploidized additive model (Add_2x_DArTSeq) using data from DArTSeq performed equally well or sometimes better than the additive model using high confidence dosage data from GBSpoly (Add_6x_GBSpoly), depending on trait architecture, for simpler quality-related traits (Fig. 4). DM had 0.33 and 0.39, Starch had 0.32 and 0.34, BC had 0.43 and 0.39, FC_P had 0.44 and 0.42, while FC_U had 0.42 and 0.35 average PA for Add_2x_DArTSeq and Add_6x_GBSpoly models, respectively (Table 4). For these traits, additive-only models were the best and the full model (Add_Non_6x_GBSpoly) always had negative PA due to a largely monomorphic relationship matrix, possibly due to the assumptions taken in calculating the additive and non-additive effects in the full model which may be complicated by the many possible combinations in autopolyploids. However, the situation changed with yield-related traits as the effects of dosage and non-additive effects became more important. For these traits, the high-quality data with dosage from GBSpoly (Add_6x_GBSpoly) were always better in prediction when compared to the additive model with diploidized data (Add_2x_DArTSeq). NOCR had 0.19 and 0.32, TNR had 0.24 and 0.38, CYTHA had 0.18 and 0.20, RYTHA had 0.18 and 0.22, and FYTHA had 0.21 and 0.24 average PA for Add_2x_DArTSeq and Add_6x_GBSpoly additive models, respectively (Table 4). However, the additive-only model with dosage (Add_6x_GBSpoly) was not always the best in PA for all yield-related traits, especially not for storage roots traits CYTHA and RYTHA, where it performed similar to either or both of the models considering non-additive effects whether with dosage (Add + Non_6x_GBSpoly) or without dosage (NonAdd_2x_DArTseq) (Fig. 4). Nevertheless, the largely monomorphic relationship matrix from the full model (Add + Non_6x_GBSpoly) ensured low predictive ability using this model for most yield-related traits as well, especially FYTHA which had the highest negative PA, (collapsed to zero in Fig. 3, for plotting purposes). In general, pseudo-diploidizing high-quality data already called with dosage (Pseudo_2x_GBSpoly) drastically reduced PA even more than using data called as diploid (DArTseq). In potato, the situation was not very different as the pseudo-diploidized additive-effects model (Pseudo_2x_GBSCornell) was the second-best model after the additive-effects-only model with dosage (Add_4x_GBSCornell) for simpler disease traits and its comparative advantage significantly reduced with more complex traits (Fig. 5). LB2014_O had 0.68 and 0.63, LB2016_Y had 0.62 and 0.52, PVY_L had 0.55 and 0.51, AYP_K had 0.45 and 0.34, WMT_K had 0.48 and 0.34, TTW16_Ica had 0.16 and 0.16, while TTW16_HLJ had 0.38 and 0.31 average PA for Add_4x_GBSCornell and Pseudo_2x_GBSCornell, respectively. As with the full model in sweetpotato, the model including non-additive effects (Add + Non_4x_GBSCornell) was the least performing in terms of PA (Table 4).

**Table 4:** Summary quantitative-genetic parameters derived from genomic selection with cross-validation applying different genetic effects models in sweetpotato and potato

Crop	Trait^a^	Model^b^	$\sigma_{a}^{2}$	$\sigma_{e}^{2}$	$h^{2}$	$PA$	$\Delta GG$
Sweetpotato	DM	Add_2x_DArTSeq	1.6935	2.9536	0.36	0.33b	0.085889
		Add_6x_GBSpoly	4.0035	2.0762	0.66	0.39a	0.176077
	Starch	Add_2x_DArTSeq	6.1716	13.7616	0.31	0.32b	0.158993
		Add_6x_GBSpoly	12.1683	11.5424	0.53	0.34a	0.265111
	BC	Add_2x_DArTSeq	150.2336	113.1697	0.57	0.43a	1.0541
		Add_6x_GBSpoly	225.1431	152.0581	0.60	0.39b	1.29041
	FC_P	Add_2x_DArTSeq	0.5416	0.3304	0.62	0.44a	0.064762
		Add_6x_GBSpoly	0.8168	0.4189	0.66	0.42b	0.081339
	FC_U	Add_2x_DArTSeq	12.9633	10.9257	0.54	0.42a	0.295238
		Add_6x_GBSpoly	16.1489	16.777	0.49	0.35b	0.305411
	NOCR	Add_2x_DArTSeq	50134102	2.93E + 08	0.15	0.19b	269.0607
		Add_6x_GBSpoly	1.36E + 08	2.43E + 08	0.36	0.32a	722.4699
	TNR	Add_2x_DArTSeq	1.86E + 08	7.40E + 08	0.20	0.24b	681.9091
		Add_6x_GBSpoly	4.71E + 08	5.83E + 08	0.45	0.38a	1606.149
	CYTHA	Add_2x_DArTSeq	8.6149	27.7157	0.13	0.18b	0.105664
		Add_6x_GBSpoly	8.6149	26.6061	0.24	0.20a	0.129145
	RYTHA	Add_2x_DArTSeq	4.6249	31.4849	0.13	0.18b	0.07742
		Add_6x_GBSpoly	10.9811	29.4989	0.27	0.22a	0.152434
	FYTHA	Add_2x_DArTSeq	7.678	26.0083	0.23	0.21b	0.116379
		Add_6x_GBSpoly	12.8721	26.6023	0.33	0.24a	0.186564
Potato	LB2014_O	Add_4x_GBSCornell	0.0189	0.0193	0.49	0.68a	0.011686
		Pseudo_2x_GBSCornell	0.0195	0.023	0.46	0.63b	0.010997
	LB2016_Y	Add_4x_GBSCornell	0.0191	0.0259	0.42	0.62a	0.010711
		Pseudo_2x_GBSCornell	0.0166	0.0323	0.34	0.52b	0.008375
	PVY_L	Add_4x_GBSCornell	0.0419	0.0738	0.36	0.55a	0.013817
		Pseudo_2x_GBSCornell	0.0364	0.0818	0.31	0.51b	0.011924
	AYP_K	Add_4x_GBSCornell	0.0118	0.0327	0.27	0.45a	0.00611
		Pseudo_2x_GBSCornell	0.0066	0.0389	0.15	0.34b	0.003453
	WMT_K	Add_4x_GBSCornell	0.0132	0.0322	0.29	0.48a	0.006893
		Pseudo_2x_GBSCornell	0.0069	0.0392	0.15	0.34b	0.00353
	TTW16_Ica	Add_4x_GBSCornell	2.00E − 04	0.0028	0.07	0.16a	0.000283
		Pseudo_2x_GBSCornell	3.00E − 04	0.0027	0.10	0.16a	0.000346
	TTW16_HLJ	Add_4x_GBSCornell	0.0061	0.018	0.25	0.38a	0.003612
		Pseudo_2x_GBSCornell	0.0049	0.0192	0.20	0.31b	0.0028

$\sigma_{a}^{2}$ is the additive genetic variation, $\sigma_{e}^{2}$ is the residual variance, $h^{2}$ is the narrow-sense heritability, $PA$ is the predictive ability, and $\Delta GG$ is the estimated rate of genetic gain considering the current breeding cycle length

^a^Traits as defined in Table 1

^b^Models: Add_2x_DArTseq = additive model using data from DArTseq called as diploid; Add_6x_GBSpoly = additive model using data with dosage from GBSpoly; Add_4x_GBSCornell = additive model using data with dosage from GBS at Cornell, Pseudo_2x_GBSCornell = additive model using data from GBSCornell with three heterozygote classes collapsed into one

### Number of markers and environments

**Fig. 6:**
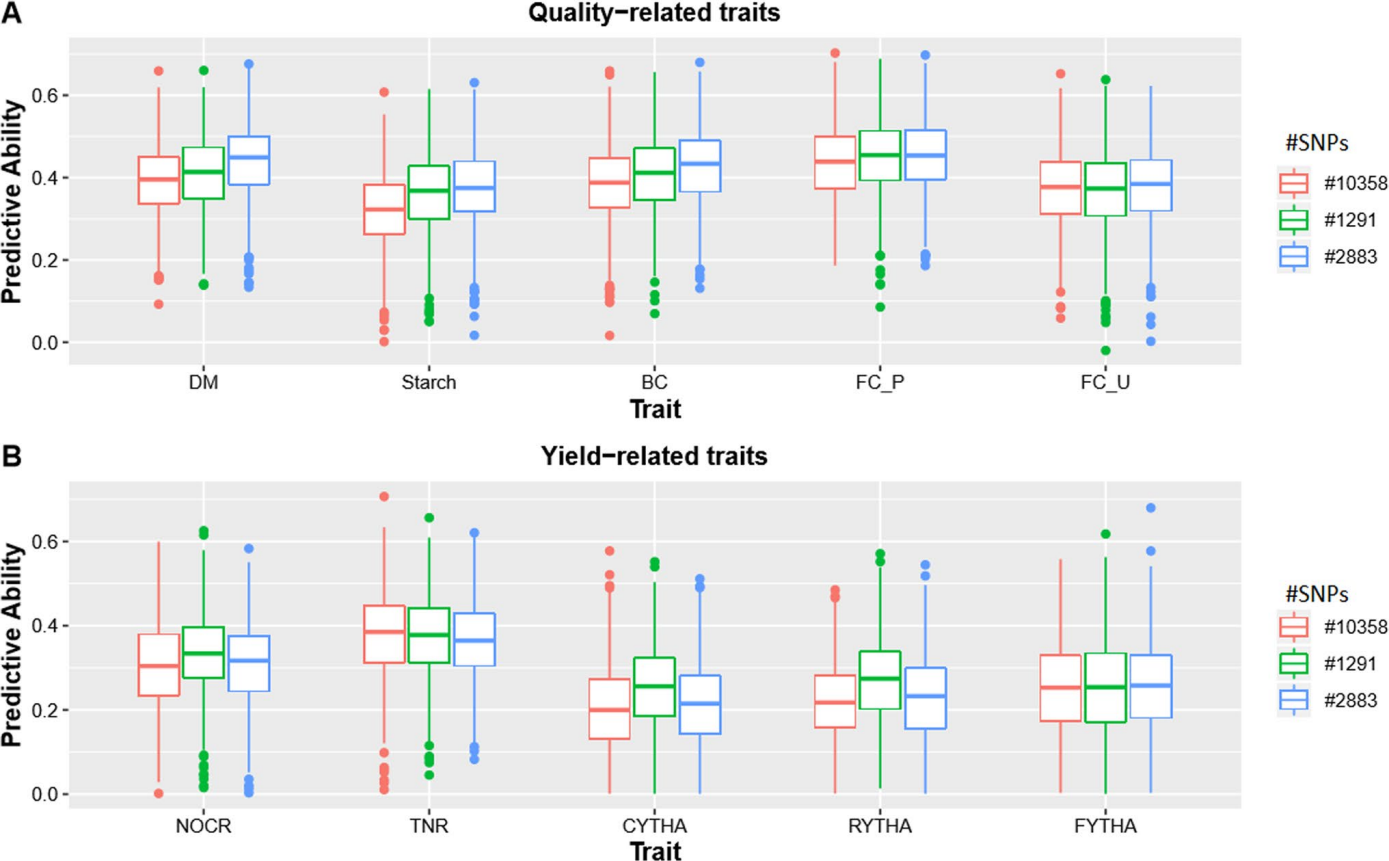
Comparing effects of allele frequency and number of markers on predictive ability in sweetpotato. Box plots comparing the effect of number of markers (different minimum allele frequency) on predictive ability using additive-effects-only model (Add_6x_GBSpoly) with 10,358 SNPs, 2883 SNPs and 1291 SNPs in sweetpotato. A; DM = dry matter, starch, BC = β-carotene, FC_P = flesh color in Peru; FC_U = flesh color in Uganda; and yield-related traits: B; NOCR = number of commercial storage roots, TNR = total number of storage roots, CYTHA = weight of commercial storage roots, RYTHA = weight of total storage roots, FYTHA = total weight of foliage in a full-sib family of sweetpotato

Our results in potato indicated that an increased number of markers by more than double did not have an effect of equal magnitude on PA (411 vs 178 SNPs; Fig. 5a vs b) considering the best predictive model (Add_4x_GBSCornell). LB2014_O had 0.69 and 0.68, LB2016_Y had 0.66 and 0.62, PVY_L had 0.59 and 0.55, AYP_K had 0.51 and 0.45, WMT had 0.51 and 0.48, TTW16_Ica had 0.19 and 0.16, while TTW16_HLJ had 0.40 and 0.38 average PA for 411 and 178 SNPs, respectively. Similarly, in sweetpotato, comparing PA using 10,358 SNPs, 2883 SNPs and 1291 SNPs using the best predictive model (Add_6x_GBSpoly) showed no effect of increasing marker density at the cost of marker informativeness (allele frequency) on PA. PA based on 10,358 SNPs which had ≥ 10% MAF generally performed lower than 2883 and 1291 SNPs which both had ≥ 30% MAF (Fig. 6). Additionally, 2883 SNPs did not have a clear comparative advantage over 1291 SNPs, both at ≥ 30% (Fig. 6), indicating that allele frequency had more effect on PA than number of markers per se, and that less informative SNPs required a higher number of markers than more informative SNPS. This was confirmed in sweetpotato by using different numbers of SNPs randomly selected from the original SNP pool having a constant MAF ≥ 5%, where 10,000 and 5000 SNPs had better PA compared to 1000 and 500 SNPs for both simple (BC) and complex (RYTHA) traits (Online Resource 10). Regarding traits in different locations, environmental effects on PA were observed which can be attributed to genotype-by-environment interaction (G x E). The PA based on the best model for FC_P (0.44; Peru) and FC_U (0.42; Uganda) in sweetpotato and LB2014_O (0.68; Peru) and LB2016_Y (0.62; China), TTW16_Ica (0.16; Peru) and TTW16_HLJ (0.38; China), in potato, were significantly different. However, the magnitude of the effect of G x E was higher for more complex yield trait, than for the simpler quality-related trait and disease trait. This can be attributed to the fact that complex traits are more prone to complex G x E interactions when compared to many simple traits.

### Effects of quantitative trait loci, haplotypes and dosage on predictive ability

**Fig. 7:**
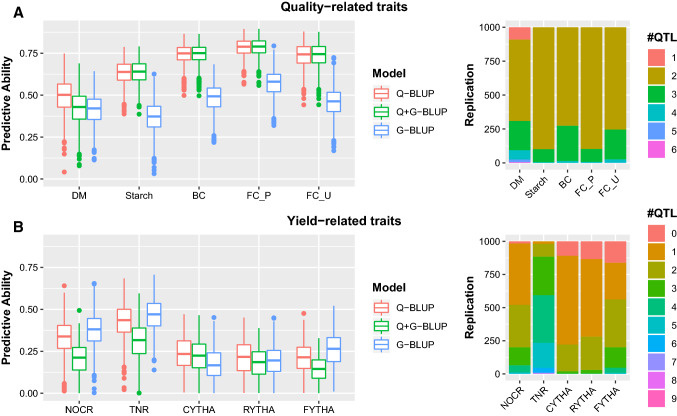
Effect of considering QTL haplotypes in sweetpotato. Boxplots comparing predictive ability of models using QTL haplotypes only in prediction (Q-BLUP); QTL combined with prediction based on markers per se, (Q + G-BLUP); prediction using markers per se without QTL (G-BLUP) for quality-related traits (A; DM = dry matter, starch, BC = β-caroten, FC_P = flesh color in Peru; FC_U = flesh color in Uganda); and yield-related traits (B; NOCR = number of commercial storage roots, TNR = total number of storage roots, CYTHA = weight of commercial storage roots, RYTHA = weight of total storage roots, FYTHA = total weight of foliage) in a full-sib family of sweetpotato. The side graphs show the number of QTL in the model at a given replication (iteration) of cross-validation following the forward QTL search and backward QTL elimination used in the random-effect multiple interval mapping method

We additionally tested three analysis models using BT sweetpotato data: (i) Q-BLUP based on relationship matrices from QTL-peak haplotypes, (ii) Q + G-BLUP fitting two terms based on QTL-peak haplotypes and the rest of the markers in the linkage map, (iii) G-BLUP, predictions using markers spaced every 2 cM in the genetic map without considering QTL. The PA results are shown in Fig. 7. Considering QTL haplotypes either per se (Q-BLUP) or with G-BLUP (Q + G-BLUP) had a clear comparative advantage for PA in simpler traits. However, this comparative advantage faded with more complex yield-related traits. Our results therefore show that with genomic selection, the comparative advantage of using the linkage map information and QTL is dependent on trait architecture, hence the magnitude of QTL effects that can be mapped (Fig. 7).

### Genetic variation, heritability and estimated rate of genetic gain

Given that the additive-effects-only model with dosage performed better for most traits in both sweetpotato and potato (Add_6x_GBSpoly and Add_4x_GBSCornell, respectively), we evaluated quantitative-genetic parameters for this model in comparison with the additive model without dosage for both crops (Add_2x_DArTseq for sweetpotato and Pseudo_6x_for potato). Narrow-sense heritability $(h^{2} )$ ranged from 0.24–0.66 for the model with dosage (Add_6x_GBSpoly) and 0.13–0.62 for the model without dosage (Add_2x_DArTseq) in sweetpotato. In potato, $(h^{2} )$ ranged from 0.07 to 0.49 in the model with dosage (Add_4x_GBSCornell) and 0.10 to 0.46 in the model with pseudo-diploidized dosages (Pseudo-2x_GBSCornell; Table 4). As expected, traits with simpler architecture (quality-related traits in sweetpotato; disease traits in potato) had the highest $(h^{2} )$ compared to more complex yield-related traits. All models across crops resulted in positive estimated genetic gain considering *L *= 5 years in sweetpotato and *L *= 8 in potato, which are the cycle lengths of current breeding schemes at CIP (Table 4). This implies that more genetic gains can be realized if such breeding cycle lengths are further significantly reduced.

## Discussion

### Low-cost, targeted amplicon sequencing platforms could realize faster genetic gains per unit time

Having a reliable, cost-efficient genotyping platform that ensures faster data turn-around to breeding programs on time to impact selection and advancement decisions is a must for routine application of genomic selection in plant breeding programs. Here we have compared results based on data from three GBS-based platforms, two of which provide data at the commercial diploid sequencing depth level (DArTSeq and GBSCornell). About 100 × read depth was required to confidently call all the five heterozygous dosage classes of sweetpotato, against 25-30x required for the diploids. These results agree with studies in potato where Uitdewilligen et al. (2013) reported that 60-80x depth was required to confidently call the three heterozygote classes. GBSpoly (Wadl et al. 2018) provided high confidence, high density SNP data which are necessary for applications requiring high density markers such as genetic linkage mapping, QTL mapping and genome-wide association mapping for trait discovery pipelines. These resources are still limited in sweetpotato. However, its density and cost are currently not amenable to routine use in plant breeding, where resources are limited. Other options for more precise genotyping such as SNP arrays, in addition to issues with ascertainment biases, are crop-specific and therefore do not benefit from economies of scale that drive costs down. Breeding programs of polyploid crops therefore have to weigh whether investing more for higher depth of sequencing is an efficient resource allocation strategy (Endelman et al. 2018). To this end, although our results show that genotype quality and consequently the number of realized SNPs are lower with low allele sequencing depth, we also show that only a small number of markers are required to realize relatively high PA, if the SNPs are highly informative. It should, however, be noted that these results may apply only to the kind of populations analyzed in the current study, and that more complex populations like large, unstructured panels may require higher density markers. These results, however, agree with the findings of Chang et al. (2019) who showed that PA can be improved by prioritizing relevant SNP polymorphisms. Similarly, Covarrubias-Pazaran et al. (2018) using three biparental populations of the American cranberry, showed that addition of SNPs after 500 markers did not result in much increase in PA as only a few hundred SNPs were needed to reach PA plateau. This therefore implies that for practical plant breeding applications, using established genotyping platforms that ensure low-costs due to scale effects and faster data turn-around will have better likelihood of success in routine application of genomic selection in polyploids. The costs associated with the need for high allele sequencing depth to confidently call SNPs could be offset by targeting only few but highly informative SNPs and investing a little more in sequencing depth of those. SNP informativeness is critical in this case, as our data show that allele frequency affects PA more than the number of markers. Guo et al. (2018) found that at allele sequencing depth between 10x and 20x, between 80 and 100 K SNPs would be required to accurately predict additive breeding values in tetraploid rye grass. Our data similarly show that at low minimum allele frequency (≥ 5%), 5000-10,000 SNPs had better PA than 500-1,000 SNPs. Since both crops already have GBS-based SNPs at high density, the process can be fast-tracked by targeting the high informative segregating loci in amplicon sequencing. This is encouraging as polyploid crops in developing countries with limited access to expensive, high-quality genotypic datasets could also deploy GS approaches.

### Modeling non-additive genetic effects has negligible contribution to predictive ability

Our results in both potato and sweetpotato show that additive-effects-only models, whether diploidized or with dosage, were comparatively better in PA than the models considering non-additive effects for all simple traits. This comparative advantage, however, lessened with more complex traits, where non-additive effects and inclusion of dosage information became slightly more relevant, although in most cases the additive-effects-only model with dosage still remained the best in terms of PA. This finding makes sense in quantitative genetic terms as the more the number of genes affecting a trait, the more the expected interaction among loci. In sweetpotato for example, issues of ‘missing’ heritability have been established for yield-related traits using the current BT population in multiple environments, where only a few QTL with very small effects were reported even though a very dense, well phased hexaploid genetic map was used (Pereira et al. 2020; Gemenet et al. 2020). According to Varona et al. (2018), the contribution of non-additive effects to genetic variance depends on the allele frequency of the causative loci, and their consideration in breeding programs can improve the prediction accuracy for breeding values and inform cross-combinations that maximize non-additive variation in progeny. Several studies have, however, shown that inclusion of non-additive effects in the prediction models has negligible effects in improving the accuracy of predicting breeding (additive) values. For instance, Endelman et al. (2018) reported uncertainty in partitioning non-additive genetic variance in tetraploid potato, whereas Crow (2010), suggested that variance due to epistasis would have little effects in plant breeding as additive variance and covariance effects quickly overshadow such contribution following selection. Non-additive effects are mainly considered important in genomic prediction (prediction for performance of different traits based on the genotype of the individual), while additive-only methods as important in genomic selection (prediction of parental value of an individual), because only additive effects can be passed from parents to progeny (Varona et al. 2018). However, our results, supported by previous findings in other crops, imply that in light of the large number of moving parts to consider, including concerns with genotyping platforms and genotype quality for polyploids, practical breeding programs for potato and sweetpotato, and perhaps other polyploid crops, will achieve more advances considering only the infinitesimal model (additive) for both genomic selection and genomic prediction.

### The relative importance of considering dosage, haplotypes and quantitative trait loci is dependent on trait architecture

Oliveira et al. (2019) showed that the relative advantage of including dosage information to PA is dependent on trait architecture. Our results confirm this and show that for simple traits diploidized data, especially when the genotypic data are directly called as diploid during variant calling, e.g., the DArTSeq data in sweetpotato rather than pseudo-diploidizing data already called with dosage, e.g., in GBSCornell data in potato, would be adequate for prediction. However, as the traits become more complex, considering dosage improves PA and therefore the rate of progress that can be made for such traits. Endelman et al. (2018) also showed that not considering allele dosage effects in potato reduced prediction accuracy by about 0.13 on average using data from the SolCAP potato SNP array, where they reported PA ranging from 0.06 to 0.63 for specific gravity, yield and fry color. Given that most traits are quantitative, we recommend the use of data with dosage that could benefit from improved genotype calling methods, such as Bayesian methods.

Our data in sweetpotato also show that for all traits, considering both QTL and haplotypes resulted in the best PA especially for simple traits, although this comparative advantage also faded with more complex yield traits. Having markers in complete LD with causative QTL for a given trait is a prerequisite for improving PA in genomic prediction (Velasco et al. 2019). The study of Cuyabano et al. (2014) showed that considering haplotype blocks rather than single markers improved PA for dairy traits in cattle. This is because haplotypes are supposed to be in tighter LD with QTL than single markers. This can be attributed to the fact that GS-only G-BLUP methods use the average genome information relationship for model building and for prediction whereas incorporating QTL analysis gives different weights (QTL effects) to different ‘significant’ genome positions (QTL positions) for model building and for prediction. Due to this, studies have proposed a combination of QTL mapping to explain trait architecture and genomic prediction, to improve PA (Spindel et al. 2016; Lopes et al. 2017; Morgante et al. 2018; Bhandari et al. 2019). Our results, however, indicate that the relative advantage of considering QTL-based haplotypes is dependent on trait architecture and directly related to the number and effect size of the QTL in question. In this case, yield-related traits did not show much improvement in PA when QTL were considered. Despite this finding, additional efforts in studying the effect of haplotype structure on PA are recommended to increase the likelihood of fully recovering the polyploid genetic information, where the information from individual dosage markers can be rather limited.

### Further considerations for optimized breeding programs using genomic selection

The PA of genomic selection is influenced by several factors including trait architecture, the size of the training population, the relationship between the training and validation populations, heritability of the trait, the level of linkage disequilibrium (LD), marker density, environmental variances and covariance among traits (Nakaya and Isobe 2012). In addition to the already discussed factors, our results indicate that genotype-by-environment interaction plays a significant role in determining PA as can be seen in the same traits measured across several environments. Additionally, PA magnitude even for simple traits was lower in sweetpotato where we used BLUEs across six environments, than in potato where predictions were made per single environment. Models incorporating genotype-x-environment interaction are important and more realistic when predicting performance of untested genotypes across environments (Burgueno et al. 2012; Heslot et al. 2014; Wang et al. 2018). Furthermore, PA for complex yield-related traits was always lower than for simpler quality-related or disease traits. PA for such complex traits has been shown to benefit from multi-trait selection models incorporating simpler, correlated traits with the primary trait (Covarrubias-Pazaran et al. 2018; Michel et al. 2019). Additionally, Bernal-Vasquez et al. (2014) alluded to the fact that phenotypic data analysis contributed to improved PA, which speaks to the necessary precision and accuracy of the phenotype in training populations. Taken together, the current results show that genomic selection will contribute toward increased genetic gains, especially via reduced breeding cycle time in potato and sweetpotato. However, the effectiveness of genomic selection will have to be considered from the perspective of optimizing the entire breeding program (Cobb et al. 2019). Therefore, given the diversity existing from program to program in terms of resources and human capacity, no ‘one size fits all’ scenario is anticipated.

Finally, it does not escape to our attention that the predictions herein are based on single populations. However, plant breeding requires several levels of allele recombination through generations. We cannot estimate from the current data, how such recombination complexity will affect the efficiency of GS in breeding programs. Additional studies estimating PA in actual multi-generation breeding populations therefore need to be carried out to reliably estimate the value of GS to potato and sweetpotato, and perhaps other polyploid breeding programs. Furthermore, we used an *F*_*1*_ population in sweetpotato and a fairly structured diversity panel in potato (Lindqvist-Kreuze et al. 2020), which may influence the number of markers required to carry out predictions. For routine application of GS therefore, there is need to determine the number of markers based on the population, its allele frequency and population structure.

## Electronic supplementary material

Below is the link to the electronic supplementary material.

Supplementary material 1 (PDF 106 kb)

Supplementary material 2 (TIFF 85 kb)

Supplementary material 3 (CSV 10709 kb)

Supplementary material 4 (CSV 2507 kb)

Supplementary material 5 (PDF 306 kb)

Supplementary material 6 (CSV 22165 kb)

Supplementary material 7 (PDF 211 kb)

Supplementary material 8 (CSV 32 kb)

Supplementary material 9 (CSV 28 kb)

Supplementary material 10 (PDF 7 kb)

## Data Availability

All single nucleotide polymorphism (SNP) data used in the current manuscript are provided with the manuscript as Online Resources 3–4, 6 while all best linear unbiased estimators (BLUEs) are provided as Online Resources 8 and 9.
