# Supplementary material for: Sequencing depth and genotype quality: accuracy and breeding operation considerations for genomic selection applications in autopolyploid crops
Source: Theor Appl Genet. 2020 Sep 2;133(12):3345–63. doi: 10.1007/s00122-020-03673-2 (PMC7567692; doi:10.1007/s00122-020-03673-2)
Supplement: Supplementary file 1 — Supplementary material 1 (PDF 106 kb) [file 122_2020_3673_MOESM1_ESM.pdf]

Sequencing depth and genotype quality: Accuracy and breeding operation considerations for genomic selection applications in autopolyploid crops

Dorcus C Gemenet[1,4], Hannele Lindqvist-Kreuze[2], Bert De Boeck[2], Guilherme da Silva Pereira[3,5], Marcelo Mollinari[3], Zhao-Bang Zeng[3], G Craig Yencho[3], Hugo Campos[2]

[1]International Potato Center, ILRI Campus, P.O. Box 25171-00603, Nairobi, Kenya
[2]International Potato Center, Av. La Molina 1895, Lima, Peru
[3]North Carolina State University, Raleigh, NC 27695, USA
[4]Current address: CGIAR Excellence in Breeding Platform, CIMMYT, ICRAF Campus, Nairobi, Kenya
[5]Current address: University of Sao Paulo, Brazil

Online Resource 1 Growing conditions for sweetpotato mapping population (BT) experiments in five environments of Peru indicating, geographic positions, elevation, mean atmospheric temperature (µTemp), mean photosynthetically active radiation (µPAR), rainfall, relative humidity, soil conditions, planting designs, planting and harvesting dates. **Published in Gemenet et al. 2020.**

	Ica16C	Ica16D	Ica17C	Ica17D	SR16
Location	Ica				San Ramon
Latitude	14° 01' 44.7" S				11°07'29"S
Longitude	75° 44' 37.5" W				75° 21' 25" W
Altitude	420 masl				820 masl
Plot size	6 m^2		4.8 m^2		9 m^2
µTemp	22 °C		24 °C		27 °C
µPAR	359 uE		458 uE		
Rainfall	0 mm		5 mm		122 mm
Relative humidity	69%			66%	61%
SoilTemp	25°C	23.6°C	27 °C	26 °C	
Soil type	Sandy Loam				Sandy clay loam
Electrical conductivity	2.26 dS/m		0.40 dS/m		0.07 ds/m
pH	7.78		8.4		5.6

	Ica16C	Ica16D	Ica17C	Ica17D	SR16
Bulk density	1.44 g/cc				
Field capacity	14.70%				
Wilting point	7.60%				
µWater potential	-233 KPa	-719 KPa	-73 KPa	-459 KPa	
Planting date	25/02/2016		15/11/2016		14/05/2016
Harvesting date	29/06/2016		17/03/2017		15/09/2016
Replications	2				3
