# Supplementary figures and images for: Sequencing depth and genotype quality: accuracy and breeding operation considerations for genomic selection applications in autopolyploid crops
Source: Theor Appl Genet. 2020 Sep 2;133(12):3345–63. doi: 10.1007/s00122-020-03673-2 (PMC7567692; doi:10.1007/s00122-020-03673-2)

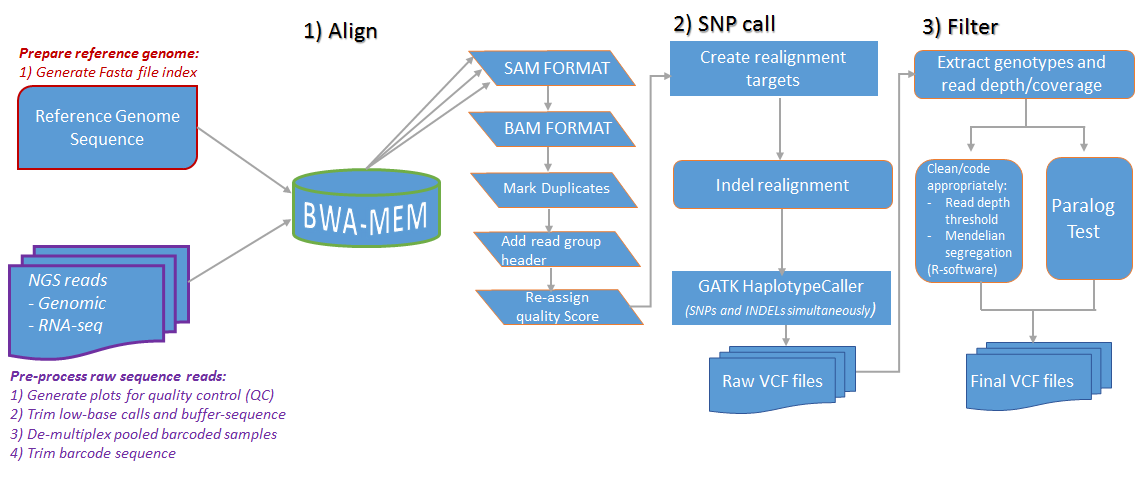

Supplement: Supplementary file 2 — Supplementary material 2 (TIFF 85 kb) [file 122_2020_3673_MOESM2_ESM.tif]

Number of markers vs PA

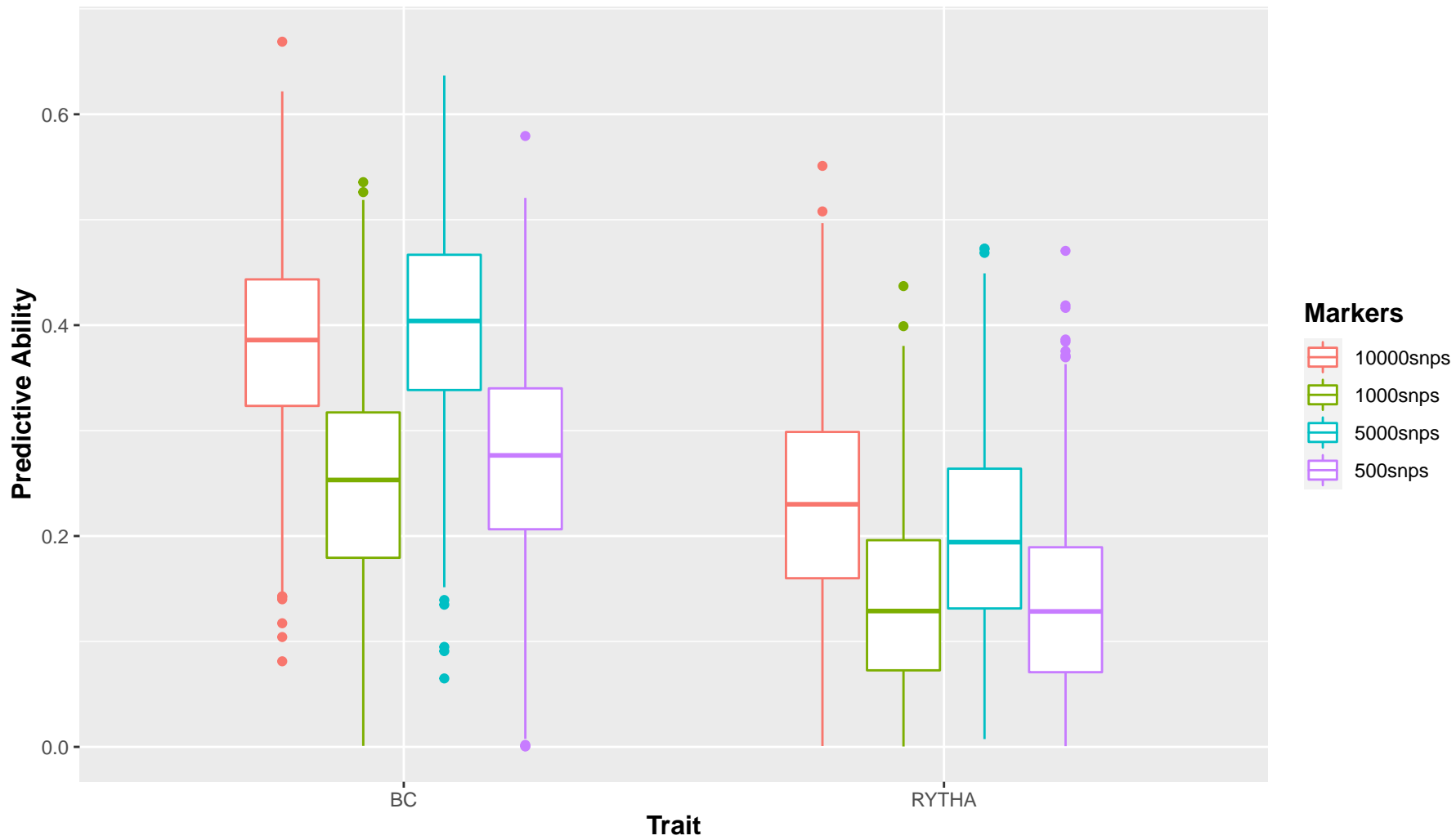

Supplement: Supplementary file 10 — Supplementary material 10 (PDF 7 kb) [file 122_2020_3673_MOESM10_ESM.pdf]
