# Supplementary material for: Sequencing depth and genotype quality: accuracy and breeding operation considerations for genomic selection applications in autopolyploid crops
Source: Theor Appl Genet. 2020 Sep 2;133(12):3345–63. doi: 10.1007/s00122-020-03673-2 (PMC7567692; doi:10.1007/s00122-020-03673-2)
Supplement: Supplementary file 5 — Supplementary material 5 (PDF 306 kb) [file 122_2020_3673_MOESM5_ESM.pdf]

Sequencing depth and genotype quality: Accuracy and breeding operation considerations for genomic selection applications in autopolyploid crops

Dorcus C Gemenet[1,4], Hannele Lindqvist-Kreuze[2], Bert De Boeck[2], Guilherme da Silva Pereira[3,5], Marcelo Mollinari[3], Zhao-Bang Zeng[3], G Craig Yencho[3], Hugo Campos[2]

[1]International Potato Center, ILRI Campus, P.O. Box 25171-00603, Nairobi, Kenya
[2]International Potato Center, Av. La Molina 1895, Lima, Peru
[3]North Carolina State University, Raleigh, NC 27695, USA
[4]Current address: CGIAR Excellence in Breeding Platform, CIMMYT, ICRAF Campus, Nairobi, Kenya
[5]Current address: University of Sao Paulo, Brazil

Online Resource 5: Segregation classes and marker frequency per class in hexaploid sweetpotato

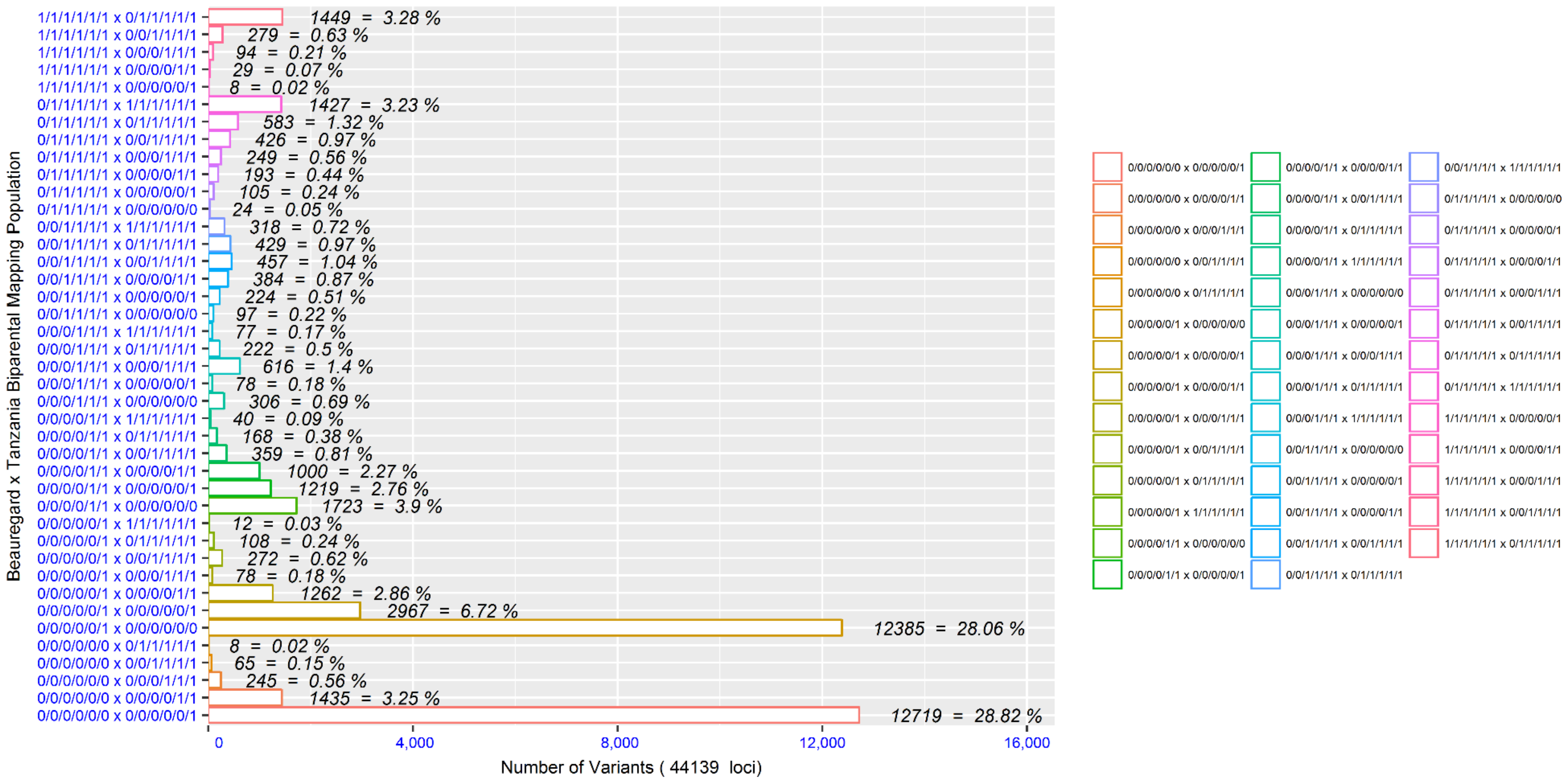

Fig. Frequency of variants in each segregation class of GBSpoly data in hexaploid sweetpotato after filtration for segregation distortion in unmethylated sites. Y-axis shows the segregation classes while the x-axis shows the number and percentage of markers in each segregation class. The expected segregation ratios are shown below.

Expected segregation ratios in hexaploid

##
###

#Filter for segregation distortion at unmethlyated loci

##

Simplex x other configuration

000001 x 000000 -> 000000 000001 -> 1:1

000001 x 000001 -> 000000 000001 000011 -> 1:2:1

000001 x 000011 -> 000000 000001 000011 000111 -> 1:4:4:1

000001 x 000111 -> 000000 000001 000011 000111 001111 -> 1:10:10:10:1

000001 x 001111 -> 000001 000011 000111 001111 -> 1:4:4:1

000001 x 011111 -> 000011 000111 001111 -> 1:2:1

000001 x 111111 -> 000111 001111 -> 1:1

#

Duplex x other configuration

000011 x 000000 -> 000000 000001 000011 -> 1:3:1

000011 x 000011 -> 000000 000001 000011 000111 001111 -> 1:6:11:6:1

000011 x 000111 -> 000000 000001 000011 000111 001111 011111 -> 1:12:37:37:12:1

000011 x 001111 -> 000001 000011 000111 001111 011111 -> 1:6:11:6:1

000011 x 011111 -> 000011 000111 001111 011111 -> 1:4:4:1

000011 x 111111 -> 000111 001111 011111 -> 1:3:1

#

```
# Triplex x other configuration
# 000111 x 000000     ->  000000    000001 000011 000111                            ->  1:9:9:1
# 000111 x 000111     ->  000000    000001 000011 000111 001111 011111 111111   ->  1:18:99:164:99:18:1
# 000111 x 001111     ->  000001    000011 000111 001111 011111 111111          ->  1:12:37:37:12:1
# 000111 x 011111     ->  000011    000111 001111 011111 111111                 ->  1:9:18:9:1
# 000111 x 111111     ->  000111    001111 011111 111111                        ->  1:9:9:1
#
# Quadruplex x other configuration
# 001111 x 000000     ->  000001    000011 000111                               ->  1:3:1
# 001111 x 001111     ->  000011    000111 001111 011111 111111                 ->  1:6:11:6:1
# 001111 x 011111     ->  000111    001111 011111 111111                        ->  1:4:4:1
# 001111 x 111111     ->  001111    011111 111111                               ->  1:3:1
#
# Pentaplex x other configuration
# 011111 x 000000     ->  000011    000111                                      ->  1:1
# 011111 x 011111     ->  001111    011111 111111                               ->  1:2:1
# 011111 x 111111     ->  011111    111111                                      ->  1:1
#
#############################################################
```
