# Supplementary material for: Sequencing depth and genotype quality: accuracy and breeding operation considerations for genomic selection applications in autopolyploid crops
Source: Theor Appl Genet. 2020 Sep 2;133(12):3345–63. doi: 10.1007/s00122-020-03673-2 (PMC7567692; doi:10.1007/s00122-020-03673-2)
Supplement: Supplementary file 7 — Supplementary material 7 (PDF 211 kb) [file 122_2020_3673_MOESM7_ESM.pdf]

Sequencing depth and genotype quality: Accuracy and breeding operation considerations for genomic selection applications in autopolyploid crops

Dorcus C Gemenet[1,4], Hannele Lindqvist-Kreuze[2], Bert De Boeck[2], Guilherme da Silva Pereira[3,5], Marcelo Mollinari[3], Zhao-Bang Zeng[3], G Craig Yencho[3], Hugo Campos[2]

[1]International Potato Center, ILRI Campus, P.O. Box 25171-00603, Nairobi, Kenya
[2]International Potato Center, Av. La Molina 1895, Lima, Peru
[3]North Carolina State University, Raleigh, NC 27695, USA
[4]Current address: CGIAR Excellence in Breeding Platform, CIMMYT, ICRAF Campus, Nairobi, Kenya
[5]Current address: University of Sao Paulo, Brazil

Online Resource 7: QTL summary information in sweetpotato

Table 1 Summary of quantitative trait loci (QTL) for dry matter (DM), starch, β-carotene (BC), flesh color in Peru (FC_P) and flesh color in Uganda (FC_U) in sweetpotato. **Published in Gemenet et al. (2020).**

Trait	QTL	[a]LG	[b]Pos (cM)	[b]SI (cM)	Score	*p*-value	σ^2_{QTL}	h^2_{QTL} [c]
DM	1	1	11.41	0.00-21.02	158.93	1.59e-04	0.6590	0.069
	2	3	37.44	4.68-62.06	604.15	<1.00e-16	3.5713	0.374
	3	3	217.13	193.17-222.06	133.93	6.00e-04	0.5904	0.062
	4	7	100.26	65.46-113.08	151.34	2.99e-04	0.5702	0.060
	5	12	150.05	146.02-155.04	264.05	1.57e-07	1.0657	0.112
Starch	1	3	37.44	0.00-84.32	849.10	<1.00e-16	21.3694	0.512
	2	12	147.31	146.02-150.05	435.34	<1.00e-16	7.0820	0.170
BC	1	3	36.14	0.00-121	1067.07	<1.00e-16	336.9740	0.502
	2	12	146.02	124.12-176.02	828.83	<1.00e-16	194.5965	0.290
FC_P	1	3	36.14	0.00-122.1	1262.29	<1.00e-16	1.1444	0.536
	2	12	146.02	117.02-180.05	944.49	<1.00e-16	0.6259	0.293
FC_U	1	3	36.14	0.00-88.10	1064.73	<1.00e-16	28.5757	0.488
	2	4	213.05	200.09-227.13	135.98	9.20e-04	1.8504	0.032
	3	12	146.02	121.41-176.02	794.68	<1.00e-16	16.2076	0.277

[a]Linkage group

[b]Position in centiMorgans

[b]Support interval in centiMorgans

[c]proportion of total variance explained by a QTL

Table 2 Summary of quantitative trait loci (QTL) for yield related traits. Obtained from **Pereira et al. (2020).**

Trait[a]	QTL	LG[b]	Pos (cM)[c]	Score	*p*-value	σ^2_{QTL}	h^2_{QTL} (%)[d]
NOCR	1	1	137.60 (99.43-152.87)	222.89	9.35×10^{-6}	0.0622	13.70
	2	3	20.18 (0.00-49.27)	172.52	1.37×10−4	0.0996	21.93
TNR	2	1	140.43 (128.08-152.87)	251.13	7.36×10−7	0.1789	10.97
	3	3	20.18 (13.11-43.69)	279.18	1.42×10−7	0.3595	22.04
	4	10	165.43 (102.26-187.27)	192.09	2.69×10−5	0.1467	8.99
	4	15	78.04 (35.50-119.08)	207.07	1.34×10−5 0	0.2131	13.06
CYTHA	1	15	5.34 (0.00-34.27)	242.24	5.62×10−6	6.3128	19.93
RYTHA	1	15	5.34 (0.00-35.50)	226.56	1.32×10−5	6.9177	18.97
FYTHA	1	10	29.09 (16.12-134.37)	203.16	4.40×10−5	2.1077	14.78

[a]Trait abbreviations: number of commercial (NOCR) and total (TNR) roots per plant, commercial (CYTHA) and total (RYTHA) root yield in $t \cdot ha^{-1}$, foliage yield (FYTHA) in $t \cdot ha^{-1}$.

[b]Linkage group

[c]Position in centiMorgans and ~95% support interval in brackets

[d]Proportion of total variance explained by a QTL.
